# Improving laminar fMRI specificity by reducing macrovascular bias revealed by respiration effects

**DOI:** 10.1162/imag_a_00249

**Published:** 2024-08-01

**Authors:** Yuhui Chai, A. Tyler Morgan, Daniel A. Handwerker, Linqing Li, Laurentius Huber, Bradley P. Sutton, Peter A. Bandettini

**Affiliations:** Beckman Institute for Advanced Science and Technology, University of Illinois at Urbana-Champaign, Urbana, IL, United States; Section on Functional Imaging Methods, Laboratory of Brain and Cognition, NIMH, NIH, Bethesda, MD, United States; Functional MRI Core, NIMH, NIH, Bethesda, MD, United States

**Keywords:** cortical layer, laminar, depth, fMRI, respiration, deep breath

## Abstract

Functional MRI (fMRI) time series are inherently susceptible to the influence of respiratory variations. While many studies treat respiration as a source of noise in fMRI, this study employs natural respiratory variations during high resolution (0.8 mm) fMRI at 7T to formulate a respiration effect related map and then use this map to reduce macrovascular bias for a more laminar-specific fMRI measurement. Our results indicate that respiratory-related signal changes are modulated by breath phase (breathing in/out or in the transition between breath in and out) during fMRI acquisition, with distinct patterns across various brain regions. We demonstrate that respiration maps generated from normal fMRI runs, such as task-oriented sessions, closely resemble those from deep-breath and breath-hold experiments. These maps show a significant correlation with the macro-vasculature automatically segmented based on susceptibility weighted imaging (SWI) and quantitative susceptibility mapping (QSM) images. Most crucially, by removing voxels most responsive to respiratory variations, we can refine high-resolution fMRI measurements to be more layer-specific, improving the accuracy of laminar fMRI analysis.

## 1 Introduction

Functional Magnetic Resonance Imaging (fMRI) (Belliveau et al., 1991) has emerged as an invaluable tool in neuroscience, allowing researchers to non-invasively study brain activity and connectivity. With the advent of ultra-high field (≥7T) human MRI scanners, the spatial resolution of fMRI has ventured into the sub-millimeter realm, enabling the discernment of functional activity and connectivity across cortical layers (Huber et al., 2017;L. Huber, Finn, et al., 2021). While fMRI resolution has been dramatically improved with the development of high field MRI, fMRI signal specificity has not been improved correspondingly with the imaging resolution. The fMRI signal is still a complex amalgamation of various physiological influences, including vascular density distribution and respiratory fluctuations.

Different fMRI measurement techniques display varying sensitivities to specific vascular components. Currently, the gradient echo (GE) blood-oxygen-level-dependent (BOLD) (Ogawa et al., 1990) contrast remains the predominant method for human functional brain mapping even at high-field high-resolution fMRI. The BOLD signal is mainly weighted toward veins and is most pronounced near large vessels. Large pial vessels on the cortical surface significantly contribute to both the BOLD signal change and the spatial bias of BOLD activation away from neuronal activity sites (Polimeni et al., 2010). fMRI measurements based on cerebral-blood-volume (CBV) and cerebral-blood-flow (CBF), such as VAPER (integrated blood volume and perfusion) (Chai et al., 2020) and VASO (vascular space occupancy) (Huber et al., 2014;Lu et al., 2005) imaging, aim to be more layer-specific by minimizing the draining vein effect present in BOLD signals. Nevertheless, in voxels containing large vessels like arteries and veins, changes in intravascular blood flow and volume still induce VAPER and VASO signal changes, thereby degrading layer specificity. Given that all these methods still measure vasculature, with BOLD being dominated by the venous component and VASO/VAPER being more arteriolar-dominated (Chai et al., 2020), they remain sensitive to local vascular properties, potentially skewing signals towards large vessels to varying extents—BOLD biased by large veins and draining vein effect, and VASO/VAPER biased by large arteries.

The influence of respiratory variations on the fMRI signal can provide insights into vascular density distribution. Minor changes in a subject’s breathing, whether in phase, rate, or depth, have shown significant correlations with fMRI signal fluctuations particularly near large vessels (Birn et al., 2006;Wise et al., 2004). Historically, these respiratory fluctuations have been perceived as artifact and noise sources, prompting extensive efforts to mitigate their impact, exemplified by the physiologic correction routine RETROICOR (Glover et al., 2000) along with respiration volume per time correction (Birn et al., 2006). However, on the other hand, the respiration-related fluctuation pattern also reflects the macro-vascular density distribution, which we can use to improve spatial specificity in high-resolution fMRI. Identifying and correcting those voxels, which are dominated by macrovasculature and most susceptible to respiratory variations, is crucial for refining the specificity of fMRI analyses to the microvasculature proximate to neural activity. This has been demonstrated as a useful strategy for conventional BOLD fMRI at low fields (<7T) (Barth and Norris, 2007;Cohen et al., 2004;Moradi et al., 2003), which might also help enhance the accuracy of laminar fMRI measurements at high fields.

This study aims to map and validate the influences of natural respiratory variations on high-resolution (0.8 mm) fMRI signals at 7T, compare these with the patterns induced by deep breath and breath-hold tasks, probe their correlation with vascular density, and utilize this respiratory variation revealed information to remove macrovascular-dominated voxels, thereby enhancing laminar fMRI specificity. While previous research mainly focused on the implications of vascular impacts on conventional resolution (≥2 mm^3^) BOLD signals at low fields (<7T), our study extends this exploration to high-field layer fMRI measurements. We acquired a series of fMRI images, using VAPER and BOLD, as well as anatomical reference images processed with susceptibility weighted imaging (SWI) and quantitative susceptibility mapping (QSM) at 7T in corresponding subjects, aiming to delineate the relationships between respiratory variations, vascular density, and fMRI signals.

## 2 Methods

### 2.1 Data acquisition

All participants (age 22–32, number of subjects listed separately for each experiment in subsequent sections) gave informed consent to participate in this study under an NIH Combined Neuroscience Institutional Review Board approved protocol (93-M-0170, ClinicalTrials.gov identifier: NCT00001360). The experiments were performed on a Siemens MAGNETOM 7T scanner equipped with a Nova single-channel transmit/32-channel receive head coil. A 3rd-order B0-shimming with three iterations was applied to the imaging region.

#### 2.1.1 Functional data acquisition using VAPER-3D-EPI

Functional images were acquired using an integrated blood volume and perfusion (VAPER) contrast (Chai et al., 2020). It combines the blood-suppression module of DANTE (Delay Alternating with Nutation for Tailored Excitation) pulse trains (L. Li et al., 2012) with 3D-EPI (Poser et al., 2010). The sequence (Fig. S1) was implemented to acquire fMRI images alternating between blood-signal-suppressed, “blood-nulled” (DANTE prepared 3D-EPI), and blood-signal-augmented, “control” (original 3D-EPI as control) conditions. Parameters of DANTE pulse train were as follows: pulse number in 1^st^/later segment = 120/18, pulse interval = 1 ms, pulse flip angle = 10°, and gradient = 25.6 mT/m (applied along x and z directions simultaneously). Image acquisition parameters were as follows: TE = 20 ms, flip angle of water excitation = 19°, 96 slices (8 slices oversampling), imaging resolution = 0.8×0.8×0.84 mm^3^, matrix size = 176×220, partial Fourier of 7/8 in both phase encoding directions, and CAIPI 3×2 (k_z_shift 1) with 2 shots per k_z_-segment (shot-selective CAIPI approach (Poser et al., 2013;Stirnberg and Stöcker, 2020)). This protocol results in a volume TR of 6.082 sec, encompassing the preparation and acquisition period of the entire 3D volume.

Through dynamically subtracting the signal in the blood-nulled condition from that in the control condition, VAPER contrast is generated to be sensitive to both cerebral-blood-volume (CBV) and cerebral-blood-flow (CBF) while BOLD weighting could be largely attenuated. Exclusively for laminar fMRI data analysis, we further correct VAPER through dynamical division by that of the control image to factor out the$\text{exp(}-TE/T_{2}^{\text{*}}\text{)}$term to remove any remaining BOLD contamination and maximize the laminar specificity. The signal of the control condition is mainly determined by BOLD contrast, thus can be treated as a conventional BOLD signal.

#### 2.1.2 Anatomical data acquisition using MT-3D-EPI with phase images

Anatomical images were acquired using a magnetization transfer (MT) weighted EPI sequence, with the EPI design identical to the functional acquisition (Chai, Li, et al., 2021;Chai, Liu, et al., 2021). This approach ensures that the anatomical and functional images are naturally matched during acquisition, allowing all analyses to be performed in native fMRI space without the need for distortion correction and anatomical-functional co-registration, thereby achieving the highest spatial accuracy in analysis.

In the human brain, GM and WM have a significant difference in the fraction of macromolecular hydrogen protons (van Gelderen et al., 2017), thus their contrast can be extracted through MT-weighted imaging and used as an anatomical EPI reference. The sequence design (Fig. S1) is identical to the functional VAPER imaging. To switch from functional VAPER contrast to anatomical MT weighting, we turned off the gradients in the preparation and maximized the RF power of the preparation pulses (FA = 10-13°, minimal RF duration allowed under the SAR limit). We also acquired interleaved images between the MT-prepared and control conditions.

The MT-weighted anatomical image was generated as$\frac{S_{CTRL}\;-\;S_{MT}}{S_{MT}}$, where$S_{CTRL}$represents the image signal in the control condition, and$S_{MT}$represents the image signal of the MT-prepared condition. This combination approach extracts the MT-saturated signal and removes the T_2_* weighting associated with the EPI readout.

For Susceptibility Weighted Imaging (SWI) and Quantitative Susceptibility Mapping (QSM) postprocessing, we also reconstructed the phase image of each MT-3D-EPI volume with an adaptive coil combination.

### 2.2 Experiment paradigm

To elucidate the relationship between respiratory variations and fMRI signal changes, we conducted experiments involving deep breath, breath hold, and visual stimuli during natural breath. Instructions and stimuli were displayed on a screen inside the scanner. Respiration was monitored using a pneumatic belt positioned at the level of the abdomen and recorded using Biopac System with a sampling rate of 500 Hz. This respiratory trace was used to validate the timing of each breath-related task and any respiratory variations. To reduce head motion, we positioned two inflatable air cushions laterally between the participants’ head and the casing of the receive RF coil.

#### 2.2.1 Deep breath experiment

Ten participants (three female) underwent the deep breath experiment (Fig. S2A). Following on-screen instructions, the subjects executed deep breaths, breathing in steadily (percentage number changing from 0 to 100% showed on the screen) over 6.082 sec (1 volume TR) and then breathing out steadily (percentage number changing from 100 to 0% showed on the screen) over another 6.082 sec. After three cycles of deep breath, subjects were instructed to breathe normally for a rest period of 36.492 sec (6 volume TRs). This trial of deep breath and rest was repeated seven times in one run. Subjects were acquainted with the deep breath task before entering the scanner.

The timing of VAPER-fMRI acquisition was adjusted so that the DANTE-prepared volume can be acquired during four distinct phases of the deep breath cycle: the breath-in period, breath-out period, and the transition periods from breath-in to breath-out and from breath-out to breath-in. Correspondingly, the control volumes in VAPER-fMRI acquisition occur in opposite breath phases. Those four scenarios were defined as breath phases of 0, 90, 180, and 270 degrees, as depicted inFigure 3A. The deep breath cycle period was designed to be exactly double the fMRI volume TR, ensuring the acquisition window aligns exactly with the deep breath cycle. Four separate runs were conducted, each aligned to one of these respiration phases.

#### 2.2.2 Breath-hold experiment

Five participants (two female) were instructed to perform an end-expiration breath-hold task structured in a block design (Fig. S2B). In each block, after a rest of 48.656-sec (8 volume TRs), participants controlled their breathing according to the instructions on the screen, first breathing in steadily over 3.041 sec, then breathing out steadily from 100 to 0% over 3.041 sec, and then holding their breath for 30.41 sec (5 volume TRs). This block was repeated 12 times in one run, with participants breathing at their own pace during rest periods.

#### 2.2.3 Laminar fMRI experiment in primary visual cortex

To explore using the pattern of respiration effects on improving laminar specificity of fMRI measurements, nine participants (three female) were asked to watch a radial checkboard visual stimulus (black/white checkerboard, contrast reversing rate 10 Hz, size 14°×10°). For six subjects, we collected data using the aforementioned 96-slices protocol. In three subjects, we tailored the acquisition to focus on the visual cortex with a slab of 26 slices (2 slices oversampling), no slice acceleration, a volume TR of 3.112 sec, and other parameters consistent with the 96-slices protocol. The stimuli were presented in a block-design manner, alternating 36.492-sec (6 volume TRs) tasks with 36.492-sec rests for 27 trials in a run. During this task run, participants breathed naturally.

### 2.3 Data analysis

#### 2.3.1 Data preprocessing of magnitude and phase images

For magnitude images, after removing the first two volumes from each run, we performed motion correction on all functional VAPER and anatomical MT data within a single session using SPM12 (Wellcome Trust Center for Neuroimaging, London, UK). Following that, we utilized the time series of fixed control and blood-nulled images from functional runs to compute the VAPER contrast, while the mean images of fixed control and MT-prepared conditions in the anatomical run generated the anatomical reference image. We excluded time points from further analysis whenever the Euclidean norm of motion derivatives exceeded 0.4 mm or when outliers from the trend comprised at least 10% of image voxels using AFNI programs (Cox, 1996).

For phase images, the first two volumes were removed from each run as done for magnitude images. We did not apply motion correction to the original phase images. For each phase image, we conducted phase unwrapping, background phase removal, SWI and QSM analysis (details inSection 2.3.2) before motion correction. Then, in the resulted images of tissue phase, SWI and QSM, we applied the motion parameters estimated from the corresponding magnitude images to them. This approach avoids the interpolation errors around phase jumps in the original phase image before unwrapping. Time points censored out in magnitude images based on motion estimations and outliers were similarly excluded in phase-related image time series.

#### 2.3.2 Vasculature segmentation

To demonstrate that the voxels related to respiration predominantly correspond to vasculature, we segmented veins and arteries based on the SWI and QSM of MT-3D-EPI images.

We first reconstructed SWI and QSM images based on the magnitude and phase image series of MT-3D-EPI control volumes before averaging across the time series. The SWI images were reconstructed using the SEPIA toolbox (Chan and Marques, 2021). The QSM images were reconstructed using STI Suite programs (W. Li et al., 2011;Wei et al., 2015). Subsequently, the magnitude, SWI and QSM images were averaged across the time series to facilitate vasculature segmentation.

Second, veins and arteries were automatically segmented from the above averaged magnitude, QSM and SWI volumes using the methodology and programs developed by (Straub et al., 2022). Briefly, this algorithm employs a shearlet-based scale-wise representation to compute a vesselness function, which is then locally thresholded to generate a vasculature segmentation. For vein segmentations, the algorithm leverages the characteristic appearance of veins—dark in SWI and bright in QSM. Comprehensive details of this method are described in (Straub et al., 2022). A consistent threshold of 0.01 on the vesselness measure was maintained as per the precedent literature (Straub et al., 2022). Given our resolution, the vessels segmented by the algorithm are macro-vessels, comprising both arteries and veins.

#### 2.3.3 Analysis of respiration-related fMRI signal changes

For the deep breath dataset, we computed the voxel-wise signal change during the stable period of the deep breath task compared to rest (Fig. S2A). Specifically, the first 12.164-sec period (2 volumes) immediately after task started was skipped to avoid transient signal changes, focusing analysis on the latter 2/3 period (4 volumes) during which the response had become more stabilized, as shown inFigure S3. The comparative rest period also excluded the initial 12.164-sec post-task period (2 volumes), analyzing only the latter 2/3 period (4 volumes) after the signal had largely returned to baseline.

In the breath-hold dataset, we computed the voxel-wise signal change during the breath-hold task period compared to the rest (Fig. S2B and Fig. S4). Specifically, we excluded the cued breath-in and out period before the breath-hold task. The comparative rest period was considered post 12.164 sec of breath-hold end (2 volumes), focusing on the subsequent 3/4 period (6 volumes) once the signal had largely returned to baseline.

**Fig. 1.**
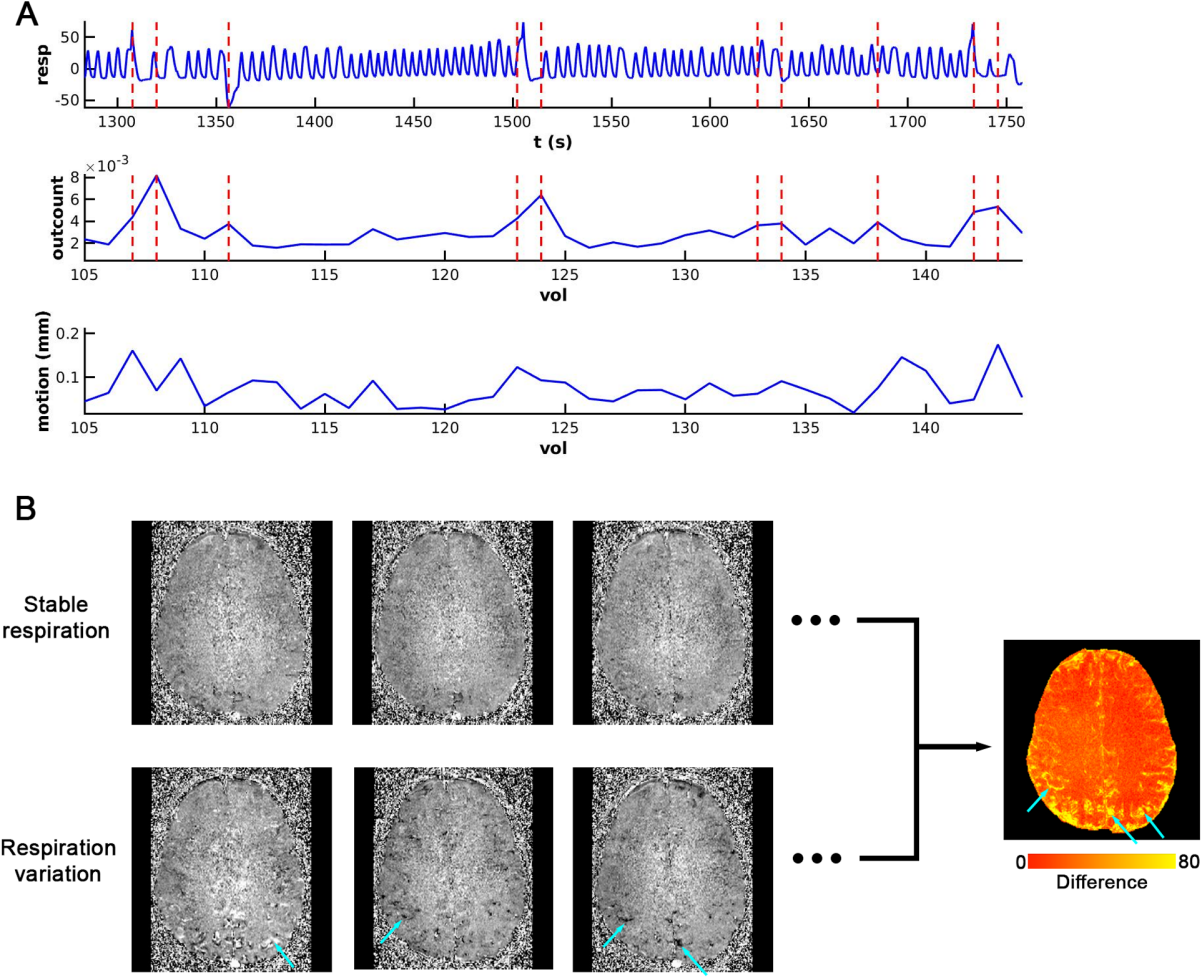
Natural respiratory variations and fMRI metrics over the same time period. (A) The first row displays the respiratory curve, measured using a pneumatic belt placed around the subject’s abdomen. The second and third rows present the outcount and motion parameters, respectively, derived from concurrent fMRI images. Vertical dashed lines mark the fMRI acquisition time points with the top 15% of outcount values. (B) The VAPER fMRI image series (raw images showing here) is divided into two subsets: the first associated with minimal respiration-related signal fluctuation during stable respiration periods, and the second associated with higher respiration-related signal fluctuations corresponding to the times indicated by dashed lines in (A). The mean absolute signal differences between these two subsets are visualized in a red-yellow overlap map on the right. Brain voxel areas most affected by respiratory fluctuations are indicated by cyan arrows.

To analyze the effects associated with natural respiratory variation, we used the dataset from the visual experiment. The AFNI program, 3dToutcount, was used to compute the fraction of voxels deviating from the signal trend at each time point. The 15% of time points possessing the highest 3dToutcount fraction were identified as the respiration variation points, with the remaining time points being considered as periods of relatively stable respiration. The absolute signal change at each respiration variation time point was then calculated relative to the mean image of stable respiration. The average of these absolute signal changes across all respiration variation time points was determined (Fig. 1B). Please note this extraction of respiration variation points from natural respiratory dataset was conducted post fMRI data preprocessing. At this stage, the time series had already been censored from the head motion and intensity outliers and we assumed that deviations in signal intensity from the trend in the preprocessed fMRI time series are primarily attributed to respiration factors. The effectiveness of this strategy is validated by the respiratory trace illustrated inFigure 1A, and the analogous respiration effect map with the deep breath and breath-hold dataset inFigure 4AB,Figure 5andFigure S5.

#### 2.3.4 Laminar analysis of fMRI response to visual checkerboard stimuli

For the fMRI visual experiment data, we conducted a general linear model (GLM) analysis on BOLD and VAPER time series which were concurrently acquired by VAPER-3D-EPI sequence. The analysis was performed with the AFNI program (Cox, 1996) 3dDeconvolve. To enable interpretation of beta weights of each covariate as percent signal changes, each voxel’s time series for every contrast was normalized by its mean signal across time before regression analysis.

We next assessed fMRI visual response across cortical depths in the primary visual cortex (V1). Cortical surfaces were reconstructed individually using FreeSurfer (Fischl, 2012) on the MT-weighted EPI images, and these were then used to determine cortical depth using the equi-volume approach (Waehnert et al., 2014) with LAYNII software suite (L. R. Huber, Poser, et al., 2021). In the context of this study, we used the term “laminar” or “layer” to indicate a measurement taken along the cortical depth, as opposed to the cytoarchitectonically defined cortical layers.

The region-of-interest (ROI) for V1 was initially defined using the Brodmann area maps obtained from FreeSurfer's reconstructed cortical surface results and subsequently transformed into volume space. The ROI was then expanded by 2 voxels to avoid any holes in the mask. We restricted the V1 ROI to areas showing BOLD activation (uncorrected p < 0.01), separating the activated regions within visual field from the deactivation (negative activation or suppression) zone outside of the visual field. This same ROI was consistently applied in both BOLD and VAPER laminar analyses.

#### 2.3.5 Node-wise correlation analysis of respiration-related fMRI variations and macrovascular density

To illustrate the spatial relationship between maps of fMRI signal changes associated with different respiratory tasks (deep breath, breath hold, and natural respiratory) and vasculature, we conducted a node-wise correlation analysis.

First, we parcellated the entire gray matter of the brain evenly into 1000 nodes using the LAYNII program LN2_COLUMNS (L. R. Huber, Poser, et al., 2021). In each node, we counted the number of voxels belonging to the type of vessels based on the vasculature segmentation, and then normalized it by the total voxel number within the node as the vessel density. We also computed the mean respiration-induced signal changes for natural breath, deep breath, and breath-hold datasets separately. The correlation coefficient was then computed between each pair of respiration effect maps and between the respiration effect and vascular density, collating data from all nodes and subjects. To account for individual differences in the respiration effect and vessel density, we normalized the respiration effects and vessel density of each node by the mean across all nodes in each brain before conducting the correlation analysis across the collective dataset.

To display the relationship between different respiration effects and vascular density, we calculated two-dimensional (2D) histograms to show how the 1000 nodes are distributed in the domains of respiration effect strength and vascular density. Each cell in the 2D histogram plot counts the number of nodes at a given vascular density and respiration effect strength. The fitted curve was also added to the plot together with the corresponding correlation coefficient values.

#### 2.3.6 Evaluation of laminar specificity and spatial blurring before and after removing respiration effect dominated voxels

To demonstrate that the fMRI map of natural respiration variation effects can be used to improve laminar specificity of fMRI measurements, we extracted the laminar profiles of BOLD and VAPER response to visual stimuli, before and after removing the top 10% of voxels most responsive to respiration. It is known that visual input from the thalamus targets the middle layer of V1 during contrast-reversing stimulus (Chen et al., 2013;Jin and Kim, 2008), while fMRI responses typically show a bias towards the cortical surface (Chai et al., 2024;Huber et al., 2017;Kim and Ogawa, 2012). The improvement of laminar specificity to sites of neural activity can be determined based on the reduction in this cortical surface bias and the alignment of the profile peak closer to the middle layer, which corresponds to the primary cortical depths of neural activity.

In addition to comparing laminar profiles, we also evaluated the fMRI spatial blurring before and after removing those respiration effect dominated voxels (Fig. S7). Spatial blurring was assessed as the mean correlation of each voxel’s time series with its neighbors using LAYNII program LN_NOISE_KERNEL (L. R. Huber, Poser, et al., 2021) over the whole FOV or V1, respectively, and then averaged across all sessions. Profiles were extracted from the signal blurring kernel of VAPER images along each spatial axis (x, y and z) across the center, and the full width at half maximum (FWHM) of the blurring kernel was computed in x, y, and z directions.

## 3 Results

Figure 1illustrates the respiratory wave and VAPER-fMRI metrics captured during a segment of the fMRI visual experiment run. By analyzing the fraction of voxels diverging in signal intensity from the trend (outcount inFig. 1A) at each time point, we identified the top 15% of time points with the highest outcount fraction as the respiration variation points. These time points are represented by vertical dashed lines overlaying the respiratory and outcount wave, denoting instances where respiratory variation occurs within the acquisition time of each fMRI volume. Additionally, the Euclidean norm of head motion derivatives is plotted over the same timeframe, all falling below the motion censoring limit (0.4 mm). The signal change at each respiration variation time point (marked by vertical dashed lines) was calculated relative to the mean image of the remaining stable respiration time points. Given the variability in signal change at different respiration variation time points, including the positive or negative sign and amplitude, we computed the absolute value. The mean of these absolute signal changes across all respiration variation time points is represented inFigure 1B.

**Fig. 2.**
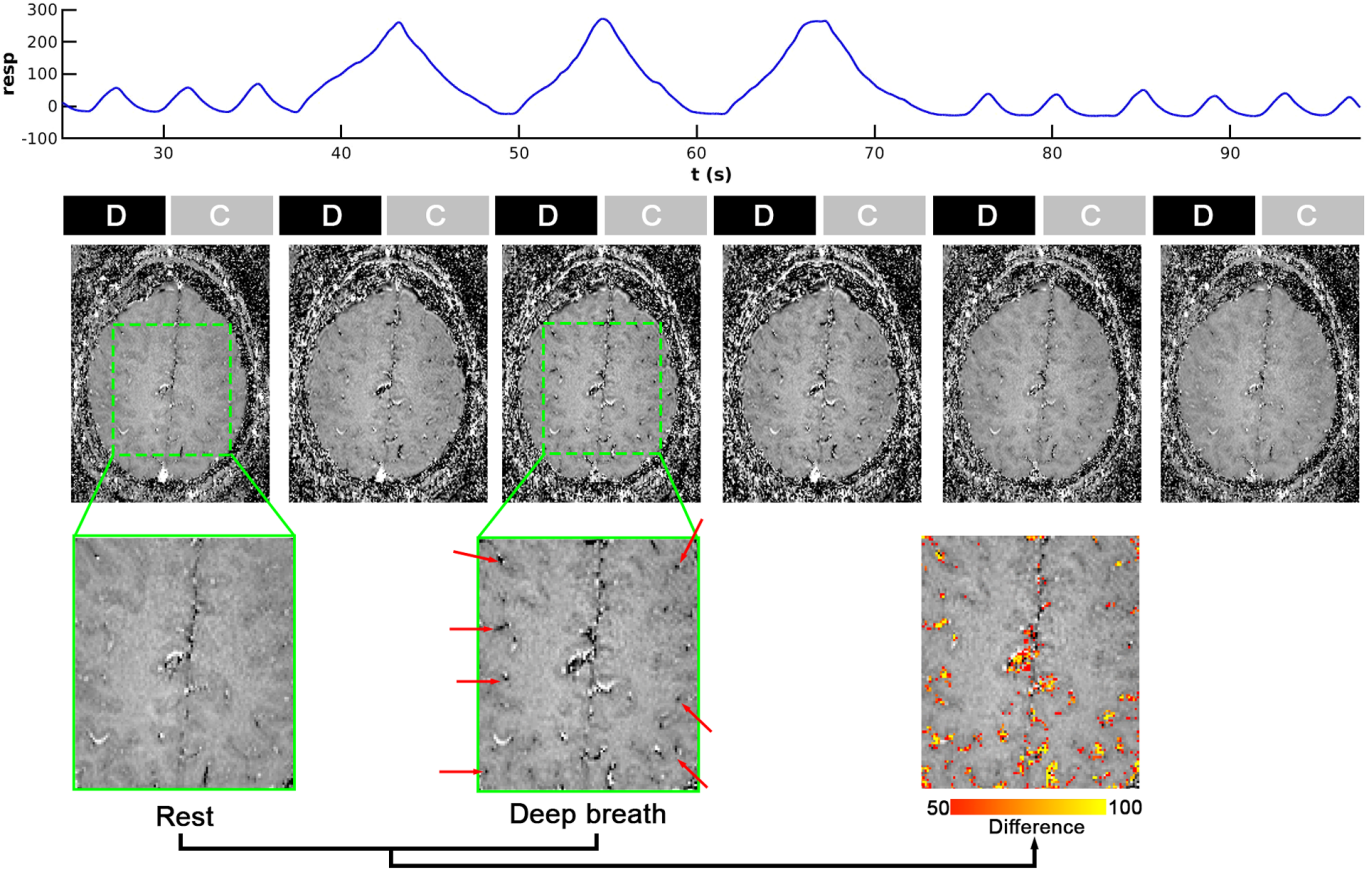
Respiration curve and VAPER images captured during the deep-breath experiment. The respiration curve is extract from a single block period, which starts with a rest period featuring natural breath, followed by three cycles of deep breath, then returns to a rest phase with natural breath. Corresponding to the same time axis of respiratory curve, the black and gray shaded boxes under the time axis represent the interleaved acquisition of DANTE-prepared EPI and Control volume acquisition, abbreviated as “D” and “C,” respectively. Below this, the mean VAPER images during the same block period are displayed. Voxels displaying the most significant changes due to deep breath are highlighted with red arrows. These signal changes induced by the deep breath are further visualized in a red-yellow overlay map.

To determine whether the variations in the fMRI signal outcount fraction are indeed induced by respiration and to understand how these variations are affected by the relative timing between VAPER-fMRI acquisition window and breath phases, we conducted a deep breath experiment. In this experiment, the subjects’ breath depth was significantly increased, and the fMRI acquisition window center was modulated to occur at different breath phases.Figure 2illustrates the respiratory wave, VAPER-fMRI acquisition, and images over one block period of deep breath experiment. Signal changes induced by deep breath are most pronounced in the voxels dominated by large vessels, as indicated by the red arrows.

**Fig. 3.**
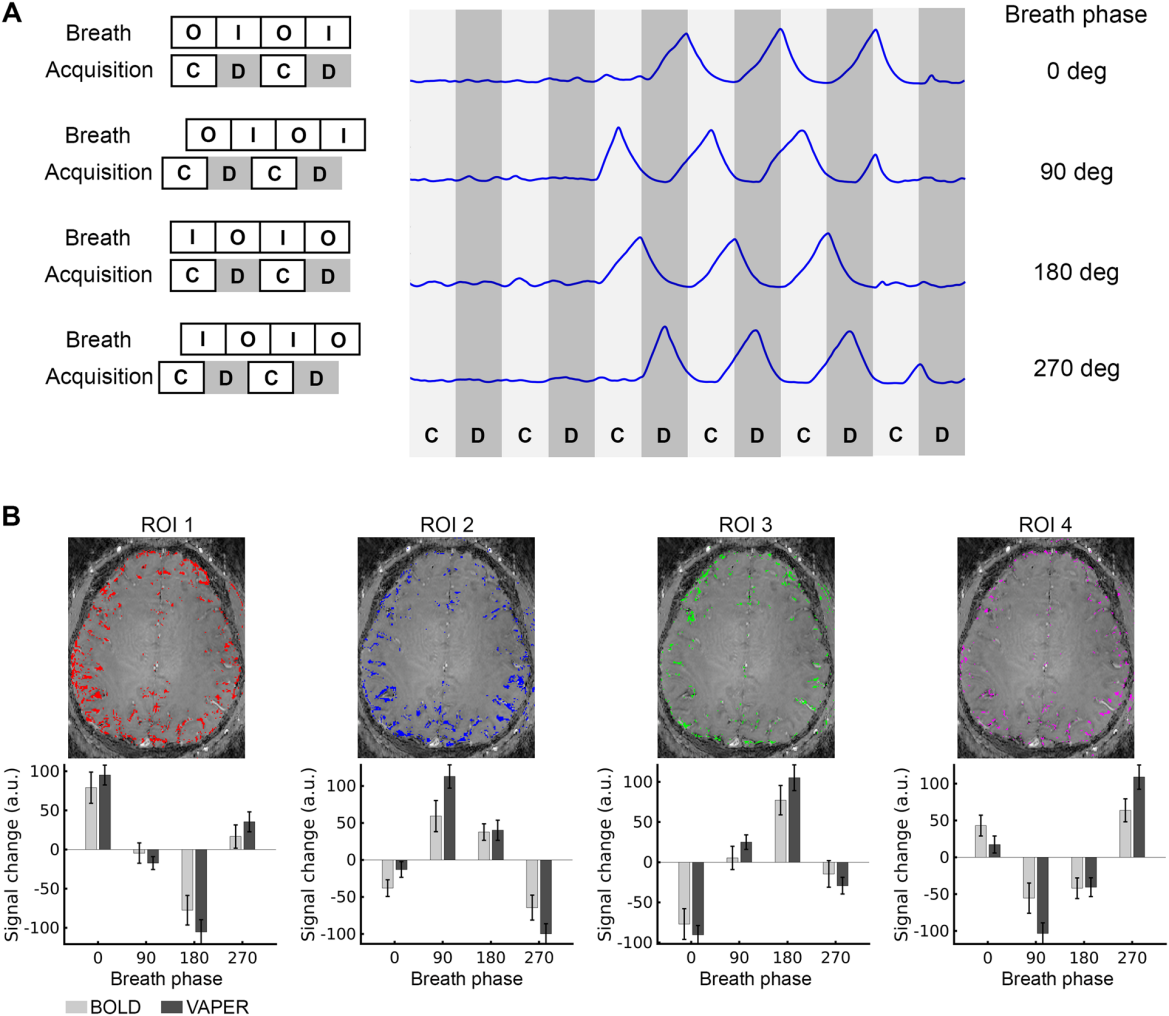
Signal changes in VAPER and BOLD images according to different phases of deep breath. (A) VAPER images were acquired at four distinct phases of deep breath across different runs: • 0 degree, where Control (“C”) volumes are acquired during breath-out (“O”) and DANTE-prepared EPI volumes (“D”) during breath-in (“I”); • 90 degree, where “C” is acquired during the transition from breath-in to breath-out and “D” during the transition from breath-out to breath-in; • 180 degree, where “C” is acquired during breath-in and “D” during breath-out; • 270 degree, where “C” is acquired during the transition from breath-out to breath-in and “D” during the transition from breath-in to breath-out. (B) fMRI signal changes induced by deep breath at different respiration phases. Four region-of-interest (ROIs) were defined based on the phase of deep breath that resulted in maximum signal change. In ROI1, deep breath at phase 0 degree led to maximum positive signal change, while a phase of 180 degree yields maximum negative signal change. Phases at 90 and 270 degree resulted in near-zero signal changes. These signal changes at different breath phases follow a sinusoidal-like curve, with a quarter-cycle shift in ROI2, half-cycle shifts in ROI3, and three-quarter-cycle shifts in ROI4. Error bars represent ± SEM across 10 subjects for VAPER and 5 subjects for BOLD. BOLD data here were acquired separately for comparison, by switching off the DANTE preparation but maintaining all acquisition, stimuli, and analysis same with VAPER data.

Figure 3Aillustrates the shifting of the VAPER-fMRI acquisition onset relative to the onset of the deep breath cycles. For comparison purposes, BOLD-fMRI was also acquired in separate runs during same deep breath tasks, by deactivating the DANTE preparation but maintaining consistent acquisition parameters and analysis with VAPER data. The observed fMRI signal changes varied across different voxels in response to the deep breath task at these phases. To better characterize this variation, four region-of-interest (ROIs) were defined based on the phase of deep breath that resulted in maximum signal change. Specifically, ROI1 included voxels showing the maximum positive signal change at phase 0 degree; ROI2 included voxels with peak positive change at phase 90 degree; ROI3 included voxels showing maximum positive change at phase 180 degree; and ROI4 included the voxels showing maximum positive change at phase 270 degree. As a result, these ROIs were broadly distributed across the brain and did not cluster in any specific area, as shown inFigure 3B. For the same deep breath phase, such as 0 degree, the signal change in ROI1 is most positive, whereas near zero in ROI2 and ROI4, and most negative in ROI3. These signal changes across varied breath phases manifest a sinusoidal-like curve, with a quarter-cycle shift in ROI2, half-cycle shifts in ROI3, and three-quarter-cycle shifts in ROI4. This signal change pattern is consistent in both VAPER and BOLD, with no significant difference (two-sample t-test, t(13) < 2, p > 0.05) in the signal change amplitudes across all breath phases and ROIs.

**Fig. 4.**
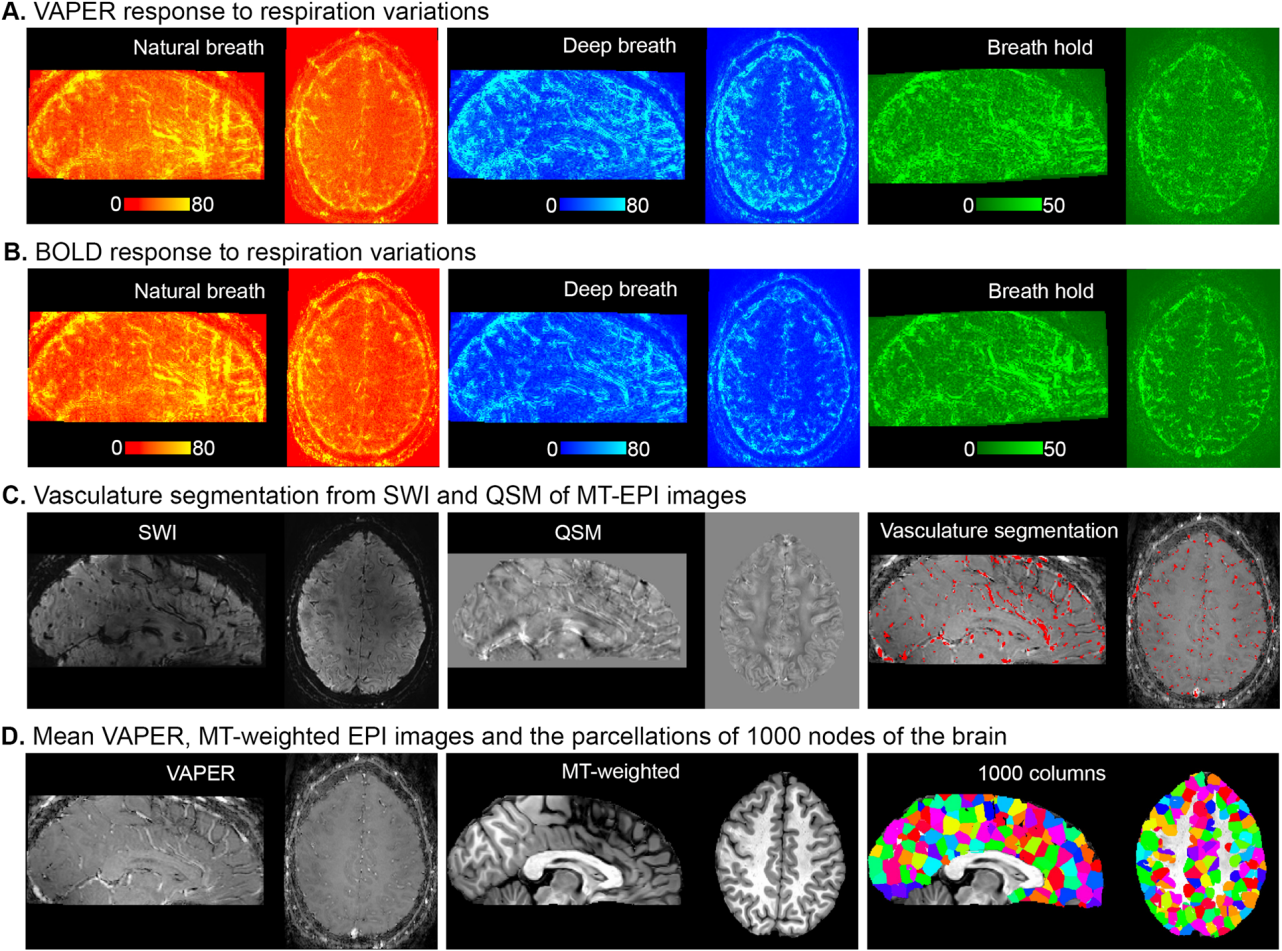
Visualization of fMRI signal fluctuations in response to diverse respiratory variations, vasculature segmentations, and varying contrast images. (A) Brain maps of VAPER absolute signal changes induced by natural respiration variation, deep breaths, and breath holds. (B) Brain maps of BOLD absolute signal changes induced by natural respiration variation, deep breaths, and breath holds. The spatial distribution patterns are very similar across diverse respiratory tasks with distinct fMRI signal contrast. (C) Vasculature segmentation derived from the SWI and QSM analysis of MT-EPI images. (D) The mean VAPER and MT-EPI images, with the cerebral cortex partitioned into 1000 nodes based on the MT-EPI image.

To validate whether the fMRI signal change patterns observed during natural respirations inFigure 1truly represent respiration-related effects, we compared them with signal change patterns induced by deep breath and breath-hold tasks.Figure 4Adisplays VAPER maps that elucidate absolute signal changes induced by various respiratory variations. The significant correlations between these maps (r = 0.77, 0.78 and 0.63), depicted inFigure 5A-C, substantiate the effective extraction of fMRI maps related to natural respiratory variations. Analogously, the BOLD response to different respiratory variations (Fig. 4B) maintains significant inter-correlations, as seen in Figure S5A-C.

To demonstrate that the respiration effect on fMRI is significantly related to the brain vasculature, we segmented vein and arteries using SWI and QSM of MT-3D-EPI images and evaluated the relationship between the macrovascular density and respiration-related effect patterns.Figure 4Cillustrates vasculature segmentation resulting from SWI and QSM analysis of MT-EPI images, andFigure 4Ddelineates the mean VAPER and MT-EPI images, with the cerebral cortex subdivided into 1000 columns/nodes based on the MT-EPI image. Subsequently, the node-wise correlation between the maps of VAPER and BOLD fMRI signal changes, invoked by various respiration variations, and vascular density were computed as inFigure 5andFigure S5, all exhibiting significant correlations (all r > 0.36). In comparison to the correlation between different respiration effects, these correlation coefficient values are relatively smaller, likely due to two reasons: (1) The missing of macrovessel segmentation around the brain edges—since QSM reconstruction masked the regions outside the brain—inadvertently omitted some large vessels located at the cortex’s periphery; (2) The vessel segmentation algorithm was only partially successful in detecting macrovessels, leaving a significant portion of them undetected. As an experiment, we applied the same vessel segmentation algorithm but with a low threshold strategy. The resulting vessel segmentation map demonstrates a significantly higher correlation (all r > 0.54) with the respiration effects, as illustrated inFigure S6.

**Fig. 5.**
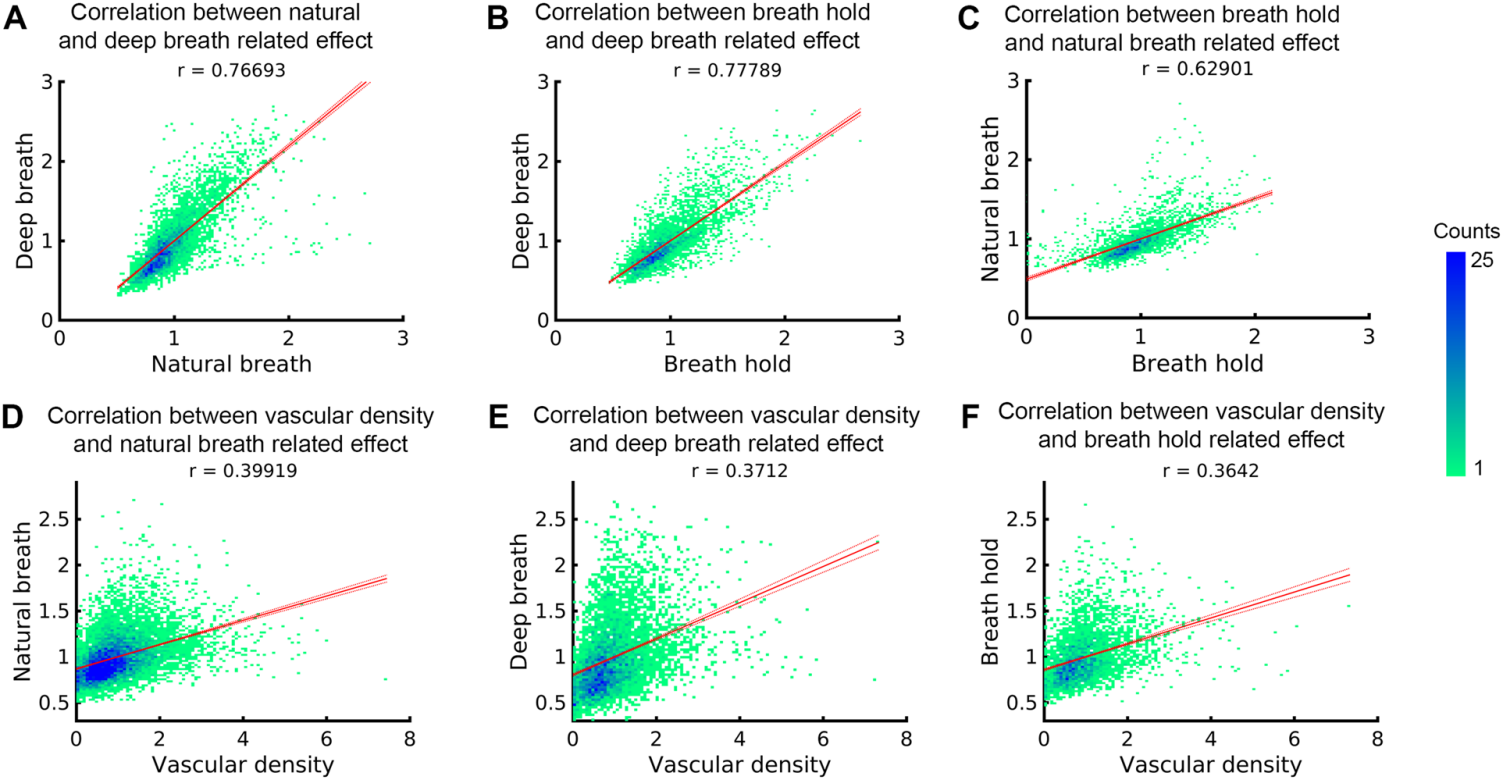
Examination of the node-wise correlation of VAPER signal fluctuations induced by various respiratory variation tasks and their association with vascular density. (A-C) Node-wise correlation assessments for natural respiratory variation, deep breath, and breath-hold induced signal changes. Notably, the node-wise correlation value of the map between breath hold and natural breath in (C) is lower than that in (A) and (B). Additionally, the amplitude range of the breath-hold effect on the x-axis of (C) is reduced compared to that in (B). This discrepancy can be attributed to the separate sessions in which the breath-hold and natural breath experiments were conducted. Consequently, registering the breath-hold map resulted in some blurring which reduces the amplitude range of signal change, and compromising the spatial accuracy compared to (A-B) with a same session setup. (D-F) Regional variations in respiration-induced fMRI signal changes—including those from natural breath, deep breath, or breath hold—are significantly correlated with vascular density. Please note that the correlation coefficients were computed after collating data from all nodes and subjects. To account for individual differences in the respiration effect and vessel density, we normalized the respiration effect and vessel density of each node by the mean across all nodes in each brain before conducting the correlation analysis across the collective dataset.

**Fig. 6.**
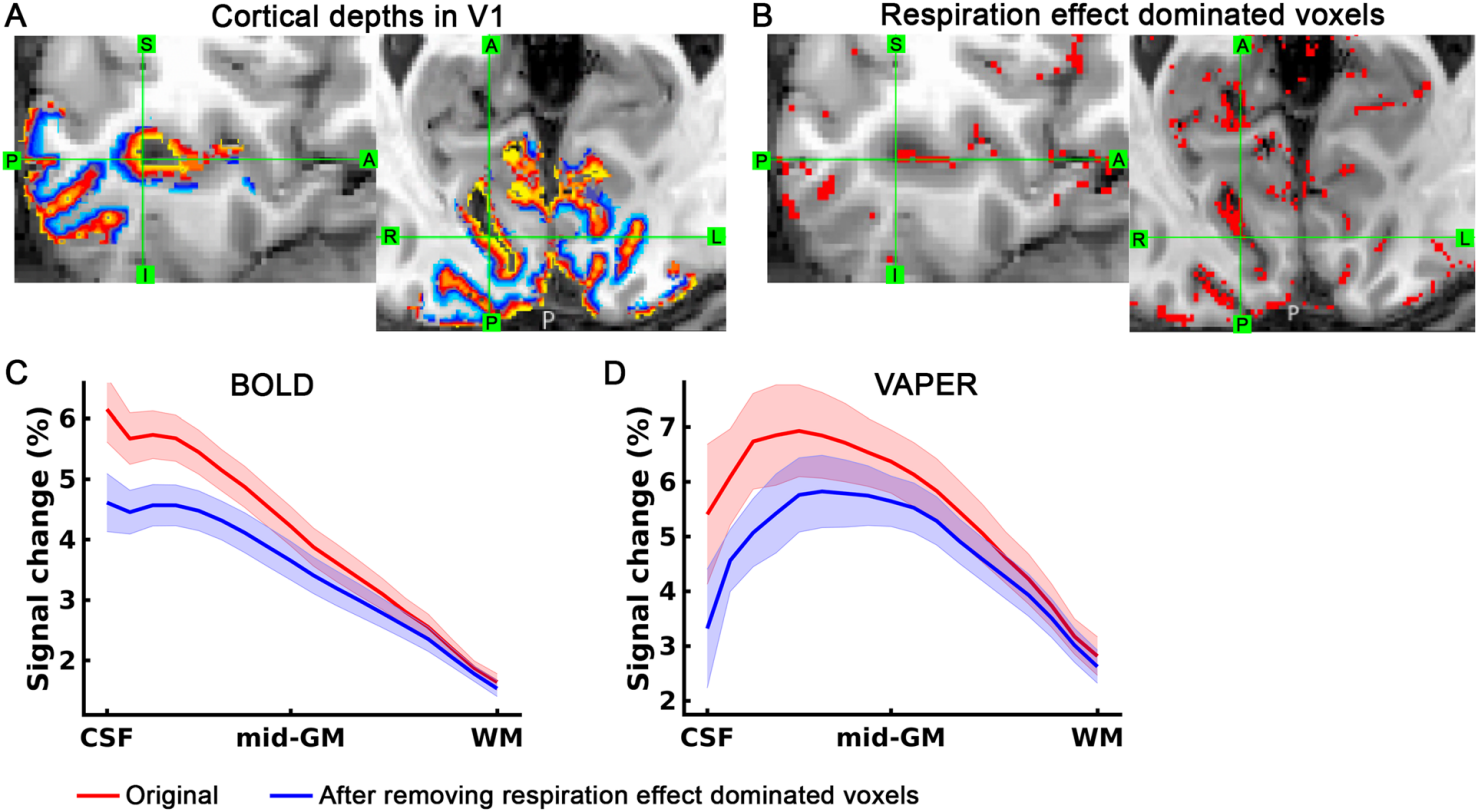
Enhancing layer-specificity in fMRI by excluding voxels most sensitive to respiration variations. (A) Cortical depths in V1 ROI for one representative subject. (B) Voxels demonstrated the strongest response (top 10% among all brain voxels) to natural respiratory variations during an fMRI visual experiment. (C) and (D) display the group-mean laminar profiles of BOLD and VAPER signal changes, respectively, induced by visual checkerboard stimuli. Red curves represent measurements extracted from all voxels across depths within V1 ROI, while blue curves show the results after removing the top 10% of voxels most responsive to respiration. On the x-axis, “CSF” indicates the CSF-GM boundary (or cortical surface), “mid-GM” represents the middle cortical depth within GM, and WM denotes the boundary between GM and WM. Shaded areas represent ± SEM across nine subjects.

To demonstrate that the fMRI map of natural respiration variation effect can be used to improve laminar specificity of fMRI measurements, we analyzed the laminar profiles of BOLD and VAPER response to visual stimuli, considering the role of respiration effect dominated voxels.Figure 6depicts that by selectively excluding voxels most sensitive to respiration variations, a refined layer-specificity in fMRI can be achieved. The cortical depth (Fig. 6Aoverlay) was reconstructed in natively fMRI space, using the MT-weighted EPI (Fig. 6Aunderlay). This analysis recognized the top 10% of voxels across the brain that are highly responsive to respiratory variations, depicted as respiration effect dominated voxels inFigure 6B. Based on the reconstructed cortical depths template, the laminar profiles of BOLD and VAPER signal changes were extracted and presented inFigure 6Cand6D. The exclusion of the respiration effect dominated voxels markedly reduced the superficial bias observed in BOLD profiles. The VAPER laminar profile, compared to BOLD, has notably less superficial bias before the exclusion of respiration effect dominated voxels (Fig. 6D). However, it is essential to note that VAPER is not entirely devoid of superficial bias due to suboptimal BOLD correction and the contribution of intravascular signals from large vessels. This residual bias in VAPER is more pronounced in some individuals (subjects 2, 7, 8 inFig. 7) and also visible at group level (Fig. 6D). Through removing those respiration effect dominated voxels corresponding to large vessels, the remaining superficial bias in VAPER can be further suppressed.

**Fig. 7.**
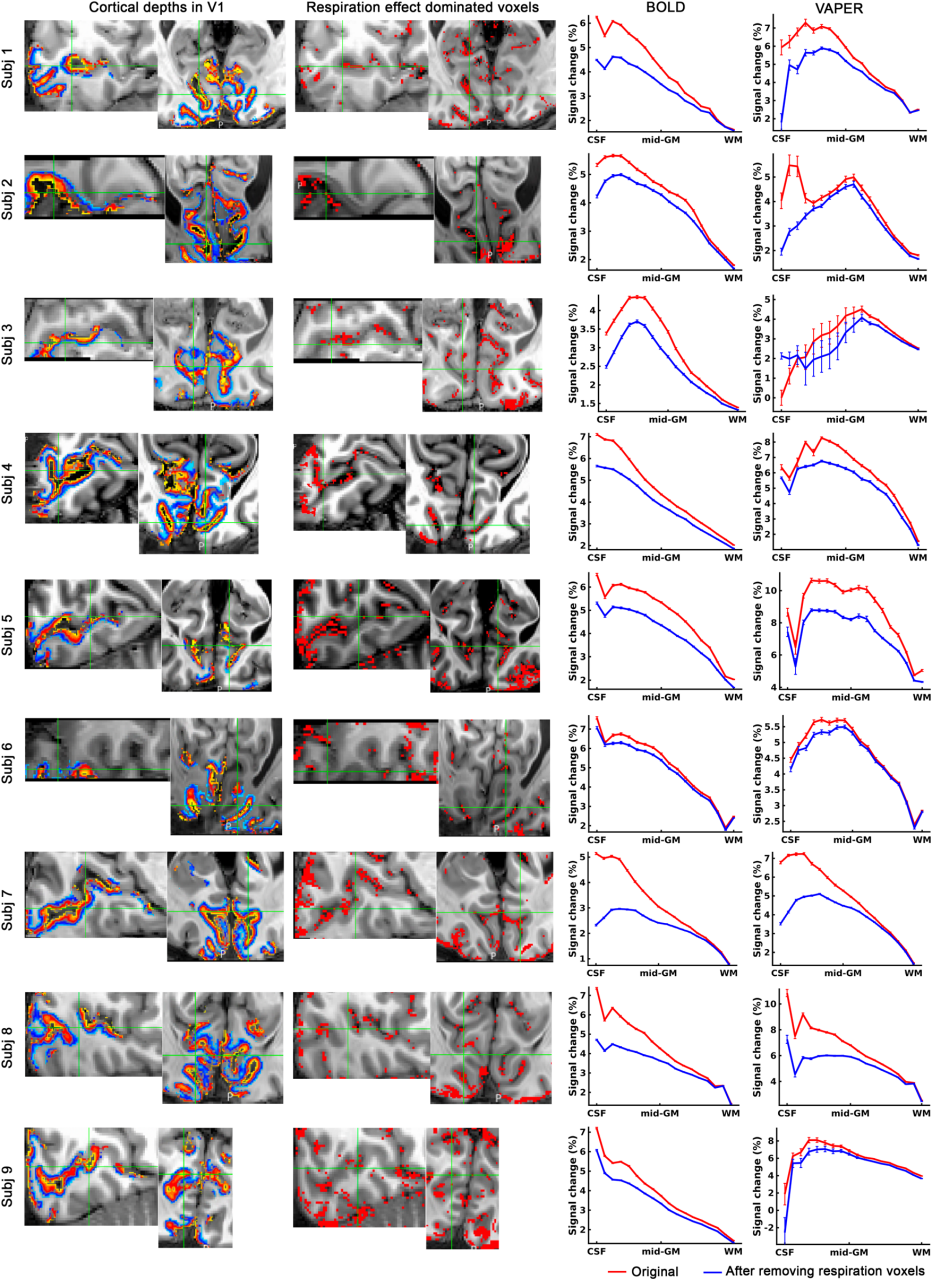
Enhancing layer-specificity in fMRI by excluding voxels most sensitive to respiration variations for each individual. Left column: Cortical depths in V1. Middle column: Voxels demonstrated the strongest response (top 10% among all brain voxels) to natural respiratory variations during an fMRI visual experiment. Right column: Laminar profiles of BOLD and VAPER signal changes induced by visual checkerboard stimuli. On the x-axis, “CSF” indicates the CSF-GM boundary (or cortical surface), “mid-GM” represents the middle cortical depth within GM, and WM denotes the boundary between GM and WM. Error bars represent ± SEM across all voxels within V1. Each row depicts results from each individual. Note, the volume TR for subject 2, 3, and 6 is 3.112 sec, while for other subjects, it is 6.082 sec.

## 4 Discussion

In this study, we explored the influence of natural variations on high-resolution fMRI signals at 7T and associated these with the signal changes induced by deep-breath and breath-hold tasks. Our findings suggested that respiration-related signal variation patterns are indicative of macrovascular density, and by excluding voxels most responsive to respiratory variations, we can improve the laminar specificity of high-resolution fMRI measurements. In the following, we will discuss the potential mechanisms underlying respiration’s influence on fMRI, its relationship with vascular structures, different strategies of using this respiration-related information to refine fMRI specificity, and limitations of current study.

### 4.1 Impact of respiratory variations on fMRI signal

Respiratory variations can influence the fMRI time series by changing the arterial CO2 level, a potent vasodilator (Birn et al., 2006). Alterations in breathing’s rate, depth, or phase can lead to variations in arterial CO2 levels, as manifested in end-tidal CO2 measurements (Van Den Aardweg and Karemaker, 2002;Wise et al., 2004). Elevated levels of CO2, acting as a potent vasodilator, contribute to increases in CBF and CBV, thus enhancing the VAPER and BOLD signal (Ogoh, 2019). Conversely, a reduction in arterial CO2 results in decreased CBF and CBV, thereby weakening the VAPER and BOLD signal. This dynamic offers an explanation for the sinusoidal-like signal shifts linked with the deep breath phase seen in our research (Fig. 3). In this study, respirations were measured using a pneumatic belt placed around the subject’s abdomen, which is easy to apply, well tolerated by all subjects, and is already in routine use for fMRI studies. The respiration belt measurement can be used as an approximation of the end-tidal CO2. Previous physiology work has shown a direct relationship between the inspired volume and the end-tidal CO2 (Van Den Aardweg and Karemaker, 2002).

Furthermore, the respiratory tasks employed in our study, including deep breaths, breath holds, and natural breath intrinsic variations, may induce pressure alterations even without a conscious effort from volunteers to escalate intrathoracic pressure. Cerebrovascular stressors like breath-holding or CO2 inhalation are known for inducing global MRI signal changes. Intrathoracic pressure changes cause rapid MRI signal alternations that have similar spatial patterns to the changes associated with breath-holding or CO2 inhalation (Wu et al., 2015). It has been shown that pressure-related fMRI signal changes also appeared in CSF (Wu et al., 2015). Alterations in blood pressure result in subsequent changes in CBF and CBV, which leads to different partial voluming effects in voxels next to CSF. This phenomenon could explain the observed respiratory variation-induced signal fluctuations around the brain edge, illustrated inFigure 4.

### 4.2 Respiratory variations as an indicator of vascular density and reactivity

Our study underscores a significant correlation between fMRI signal fluctuations during natural respiration and vascular density, as illustrated inFigure 5andFigure S5. This association is consistent with prior findings in BOLD fMRI research. For instance, earlier studies have shown that regional variations in vascular density are significantly correlated to the resting-state fMRI signal fluctuations (Vigneau-Roy et al., 2014). It is also noted that the temporal variance of the BOLD signal at rest was observed to be greater in areas around large veins (Kim et al., 1994). This observation is further validated by a separate study, which indicated that regions corresponding to larger vessels exhibited the highest standard deviation in fMRI signal over time (Birn et al., 2006).

In addition, there is substantial evidence suggesting that fMRI data, when acquired during natural respiration, inherently carry information about the cerebral vasculature (Birn et al., 2006;Wise et al., 2004). Previous studies, such as those byKannurpatti and Biswal (2008), suggest a correlation between the resting-state fluctuation amplitude (RSFA) and cerebrovascular reactivity (CVR)—a measure of cerebral vessels’ ability in dilation or constriction. BOLD fMRI has been utilized to measure CVR in response to specific tasks like breath hold (Bright and Murphy, 2013) and deep breath (Bright et al., 2009;Sousa et al., 2014). Furthermore, resting-state BOLD fMRI can also be used to assess CVR (Liu et al., 2017), leveraging innate respiratory fluctuations as an endogenous vasoactive stimulus. Our analysis resonates with these insights, emphasizing that fMRI maps, whether derived from BOLD or VAPER responses to breath hold, deep breath tasks, tend to mirror patterns observed during natural respiratory variations. Those suggest that natural respiration-linked fMRI signal fluctuations may provide insights into the vascular structure of the brain.

### 4.3 Utilization of vascular information for enhanced specificity in fMRI

In this study, we elucidated the relationship between the effects of respiration on fMRI signal and macrovascular distribution. By utilizing this insight, we can exclude respiration effect dominated voxels, enhancing the specificity of layer fMRI. The concept of leveraging the effects of respiration or macrovascular distribution to improve fMRI specificity has been explored in previous studies involving conventional resolution BOLD fMRI.

In previous fMRI analysis, one established method to mitigate the influences of the macrovasculature involves eliminating voxels whose time series coefficients of variation are much larger than the local average of their surrounding voxels (Behzadi et al., 2007). Such voxels typically coincide with regions adjacent to large vessels. Our study employed a similar strategy by removing those respiration-variation associated voxels from layer fMRI analysis for an enhanced specificity. In addition to just omitting those non-specific voxels, some researchers have sought to employ vascular information to normalize or calibrate BOLD activity. For an instance, the utilization of segmented SWI images enriched with vascular density information could assist in suppressing bias sites with large vascular contributions, thereby enhancing the spatial specificity of fMRI activation maps (Vigneau-Roy et al., 2014).

It is crucial to emphasize that the effects of vasculature are not limited to the vascular voxels themselves but also extend to adjacent areas. The influence of macrovascular structures on fMRI measurements can transcend the boundaries delineated in vascular segmentations, affecting surrounding regions, as extravascular effect from the macrovessels. Hence, instead of merely omitting voxels identified from vascular segmentations, the respiration effect map could serve as a more realistic indicator of the actual vasculature impact on fMRI specificity.

### 4.4 Limitations and areas for future research

Our study highlights the promising potential of utilizing respiratory variations to refine laminar fMRI specificity. However, there are limitations and areas that need further investigation.

Firstly, our approach primarily focused on the removal of voxels influenced by respiration. Future studies could explore alternative techniques, such as normalization or advanced modeling, to further understand and exploit this relationship. Secondly, when respiratory variations significantly correlate with fMRI tasks or there is a global change in neuronal states associated with arousal levels, emotional, or cognitive tasks—where effects are similar or spatially overlap significantly with respiration effects (Birn et al., 2009)—our approach of using respiration effects to improve fMRI spatial specificity may not be feasible. This challenge arises from the difficulty in distinguishing respiration-related effects from neuronal state related signal changes or when spatial separation is not possible. Third, our methodology does not account for the size of vessels or their specific relationship with respiration effects. We lack information about the vascular diameter of macrovessels and microvessels and how each is associated with respiration effects. This prevents us from precisely determining which diameter range of vessels are confounding factors or relevant to layer specificity. Techniques such as those used byJochimsen et al. (2010), which map vessel size in humans through hypercapnia-induced changes in BOLD effects on gradient-echo and spin-echo relaxation rates, could be adapted in future studies to assess how various vessel diameters relate to respiration effects. Furthermore, we did not measure end-tidal CO2 in this study. While our fMRI map derived from natural respiratory variations provides valuable insights, it involves complex factors such as cardiac influences, respiration related CO2 variations, and blood pressure changes. Without specific physiological measurements for each factor, isolating individual components remains challenging. Lastly, while reduced superficial bias and decreased fMRI spatial blurring suggest improvement in laminar specificity, these metrics are not as direct as measuring the point-spreading function. Future studies could benefit from a more direct assessment of spatial specificity by measuring the point-spreading function of fMRI responses in relation to the spatial extent of neuronal activity (Chaimow et al., 2018;Smirnakis et al., 2007). Additionally, evaluating the depth-dependent laminar point-spreading function could offer a more precise measure of laminar specificity (Fracasso et al., 2021;Markuerkiaga et al., 2016;Polimeni et al., 2010).

## 5 Conclusion

In conclusion, our research has illuminated the potential of natural respiratory variations as indicators for enhancing the specificity of laminar fMRI measurements. The ability to delineate and exclude voxels dominated by macrovascular effect, driven by the nuanced understanding of respiratory variations and their correlation with vascular density, represents a substantial advancement in the field of high-resolution fMRI studies.

## Data and Code Availability

Analysis code are available fromhttps://github.com/yuhuichai/respiration_layer_fMRI. The analysis pipeline uses publicly available software packages, including FreeSurfer (https://freesurfer.net/), AFNI+ SUMA (https://afni.nimh.nih.gov/), SPM (https://www.fil.ion.ucl.ac.uk/spm/), ANTs (http://stnava.github.io/ANTs/), and LAYNII (https://github.com/layerfMRI/LAYNII). The datasets can be made available from the corresponding author upon request.

## Author Contributions

Conceptualization, Y.C., A.T.M.; Methodology, Y.C., A.T.M., and D.A.H.; Formal analysis, Y.C.; Investigation, Y.C., A.T.M.; Visualization, Y.C., D.A.H.; Resources, L.L., L.H., B.P.S., and P.A.B.; Writing—original draft, Y.C.; Writing—reviewing and editing, Y.C., A.T.M., L.L., L.H., D.A.H., B.P.S., and P.A.B.; Supervision, P.A.B., B.P.S.

## Declaration of Competing Interest

The authors declare no competing interests.

## Supplementary Material

Supplementary Material
