# Supplementary material for: Improving laminar fMRI specificity by reducing macrovascular bias revealed by respiration effects
Source: Imaging Neurosci (Camb). 2024 Aug 1;2:imag-2-00249. doi: 10.1162/imag_a_00249 (PMC12272271; doi:10.1162/imag_a_00249)
Supplement: Supplementary Material [file imag_a_00249-supp.pdf]

SUPPLEMENTARY MATERIALS

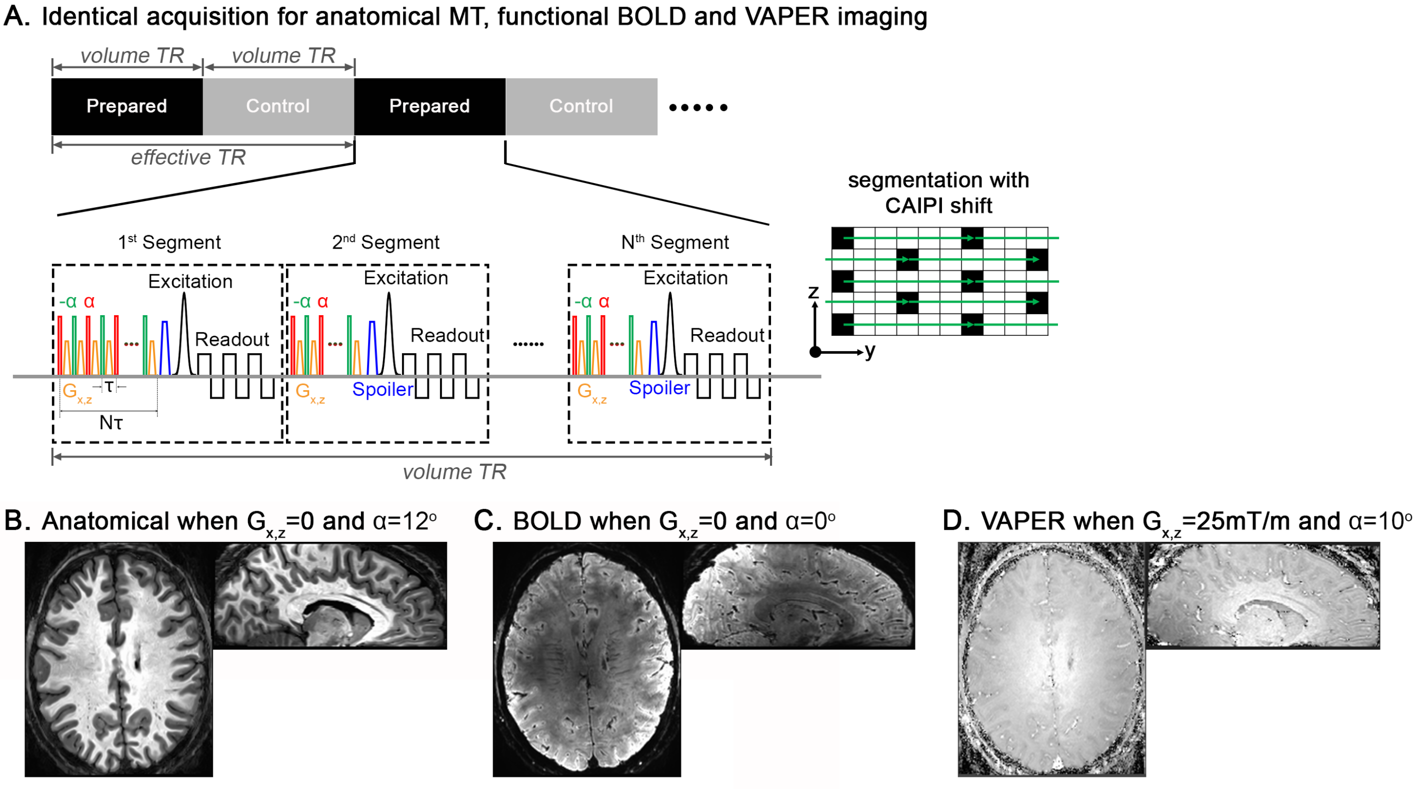

Figure S1. (A) Sequence design for functional and anatomical imaging, which utilizes an identical acquisition of shot-selective CAIPI 3D-EPI (in-plane segmentation with CAIPI shift). **(B)** Acquisition of anatomical MT-weighted images alternating between control and MT-prepared conditions. The gradients in the preparation module are switched off and power of RF pulses maximized up to the SAR limit to yield the optimal MT-weighted anatomical reference. **(C & D)** Acquisition of functional VAPER data alternating between blood-nulled module prepared and control conditions. Blood nulling is achieved by combining DANTE (Delay Alternating with Nutation for Tailored Excitation) pulse trains (turning on both gradients and RF pulses in the preparation) with 3D-EPI. In control volume, DANTE RF pulses are switched off to acquire the signal for BOLD correction.

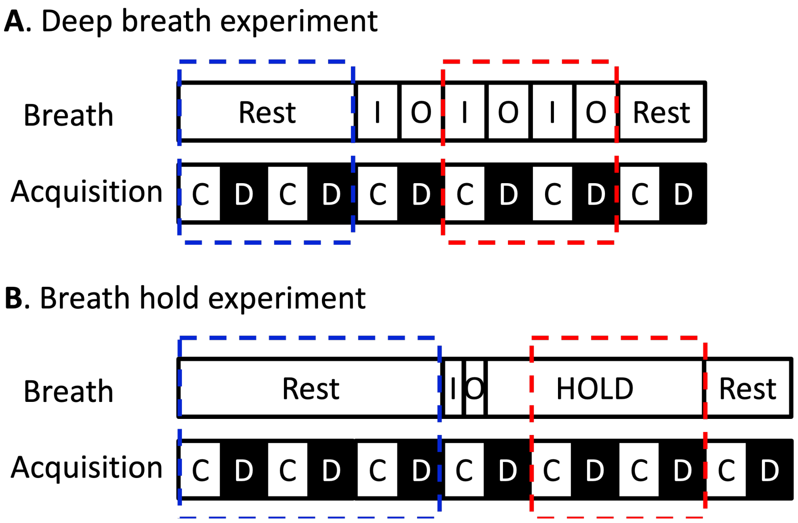

Figure S2. Experimental and analysis design for deep-breath and breath-hold tasks. **(A)** In the deep-breath task, participant was instructed to breath in ("I") steadily over 6.082 sec (1 volume TR) and then breath out ("O") steadily over another 6.082 sec. After three cycles of deep breath, subjects resumed normal breathing for a rest period of 36.492 sec (6 volume TRs). This deep-breath/rest block was repeated seven times within a single run. **(B)** For the breath-hold task, participants controlled their breathing according to the instructions on the screen, first breathing in ("I") steadily over 3.041 sec, then breathing out ("O") steadily, and then holding their breath ("HOLD") for 30.41 sec (5 volume TRs). This task block was performed 12 times in one run, with free breathing allowed during the intervening rest periods (48.656-sec, 8 volume TRs in a block). The black-shaded and white boxes in the "Acquisition" row represent the interleaved acquisition of DANTE-prepared EPI and Control volume acquisition, abbreviated as "D" and "C", respectively, both with a volume TR of 6.082 s. Signal changes were calculated by comparing the stable period during task (enclosed in in red dashed rectangles) against the rest period where the signal largely returned to baseline (enclosed in blue dashed rectangles).

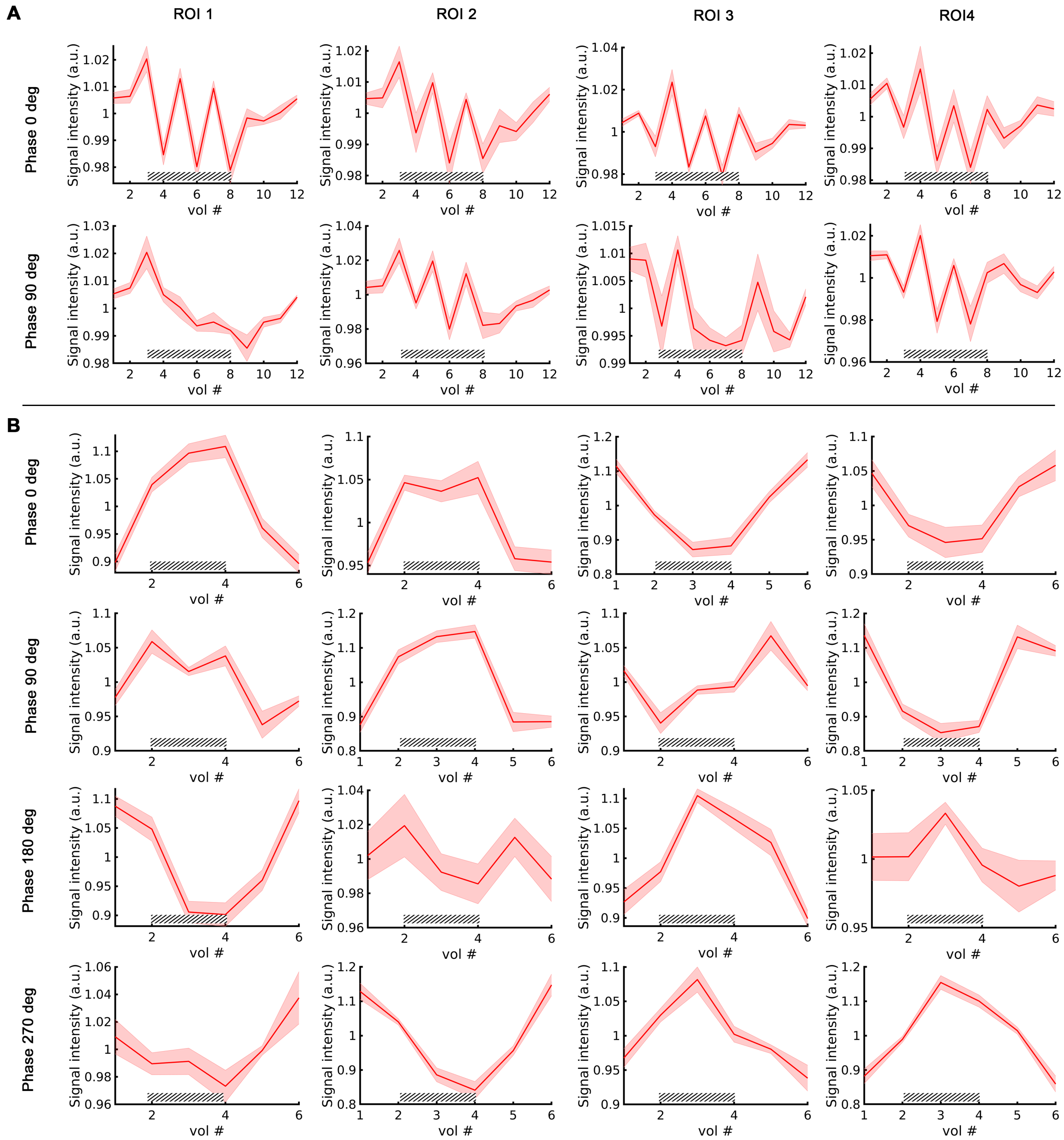

Figure S3. Group-averaged time course of fMRI response to deep breath task. The rows correspond to different respiration phases during which fMRI data acquisition occurred, and the columns represent different ROIs. Details about the respiration phases and ROI definitions can be find in Fig. 3. The slashed box under the mean time course marks the task period involving three cycles of deep breath. Time course in **(A)** is from the original BOLD signal, characterized by a zigzag pattern of rising and falling signals corresponding to either the breath-in or breath-out phase. Time course in **(B)** is from VAPER after paired subtraction. In both BOLD and VAPER, the deep-breath induced signal changes vary and even become

opposite across different ROIs and respiration phases, as detailed in Fig. 3 and Results section. Please note the respiration effect map of deep breath is generated by combining the maximum absolute signal changes across all respiration phases. All individual time courses were normalized by its mean before group averaging.

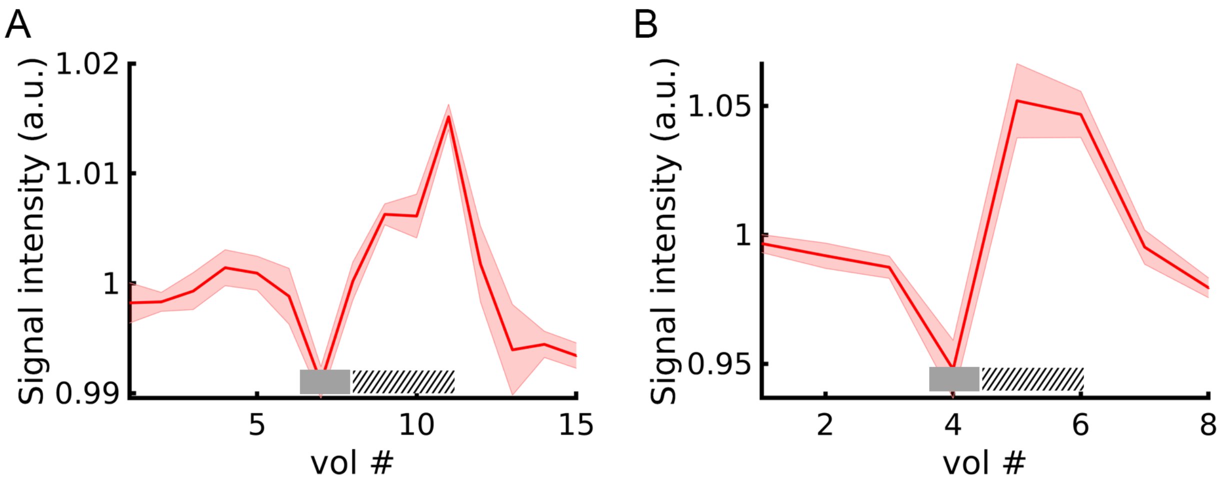

Figure S4. Group averaged fMRI response to breath hold task. **(A)** Mean time course of BOLD response. **(B)** Mean time course of VAPER response. The gray boxes under the mean time courses mark one cycle from breath-in to breath-out, while the slashed boxes indicate the periods of end-expiration breath hold.

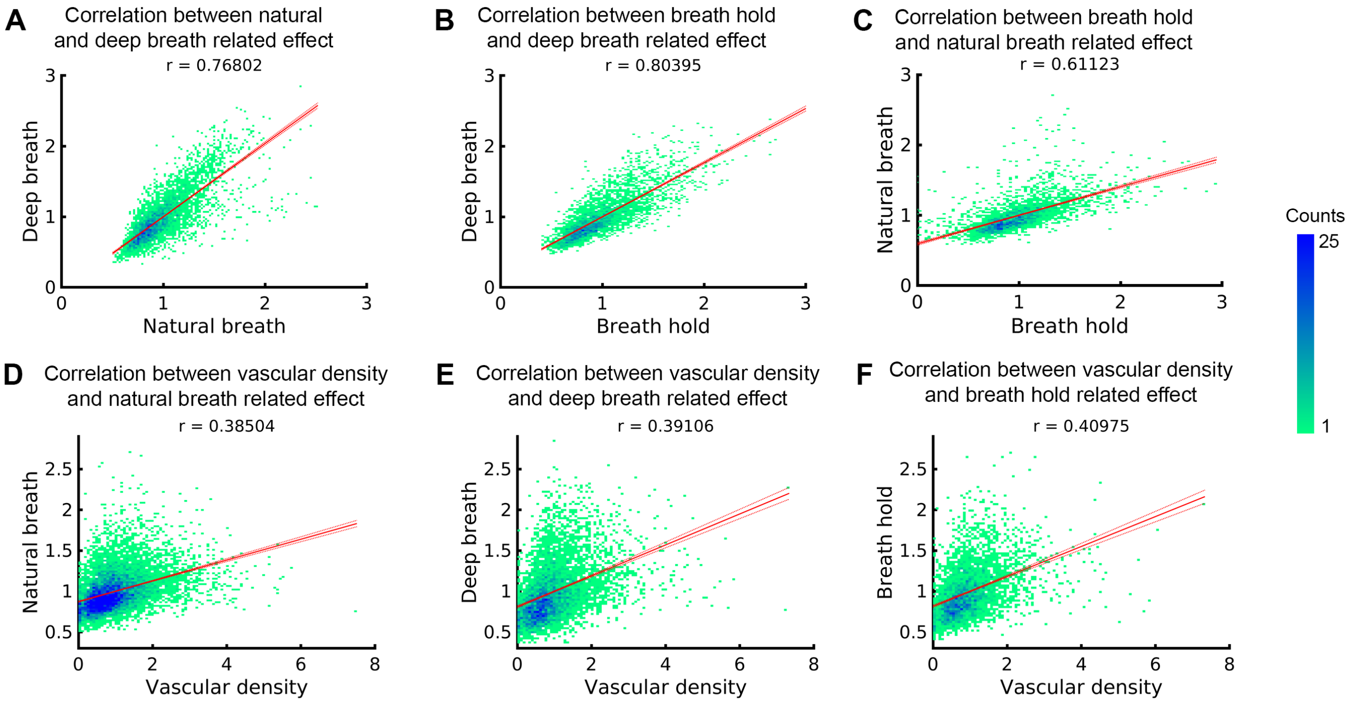

Figure S5. Examination of the node-wise correlation of BOLD signal fluctuations induced by various respiratory variation tasks and their association with vascular density. **(A-C)** Node-wise correlation assessments among natural respiratory variation, deep breath and breath hold induced signal changes. **(D-E)** Regional variations in respiration-induced fMRI signal changes—including those from natural breath, deep breath or breath hold—are significantly correlated with vascular density.

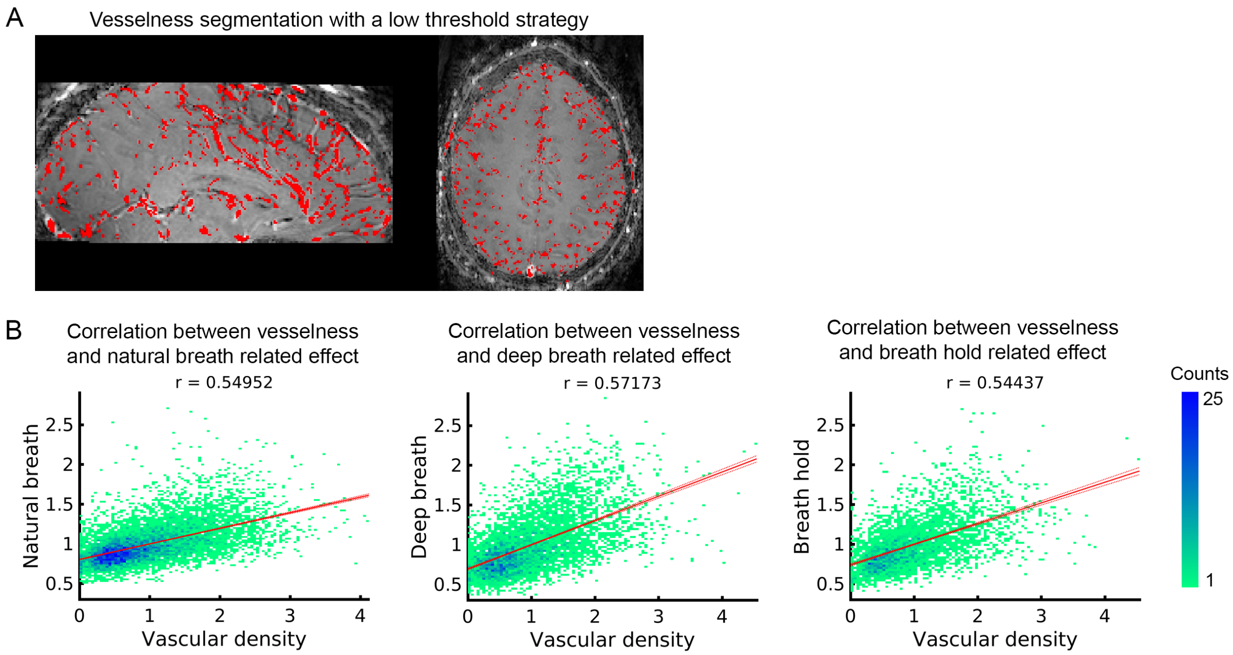

Figure S6. (A) Vesselness segmentation map using a low threshold strategy. Unlike the vessel segmentation presented in Fig. 4C of the main text, which follows a standard threshold as per Straub et al. (2022), this vesselness segmentation employs a less stringent threshold strategy (script available at https://github.com/yuhuichai/respiration_layer_fMRI/blob/main/vessel_seg_chai.m). **(B)** Correlation with respiration effects across different respiratory tasks.

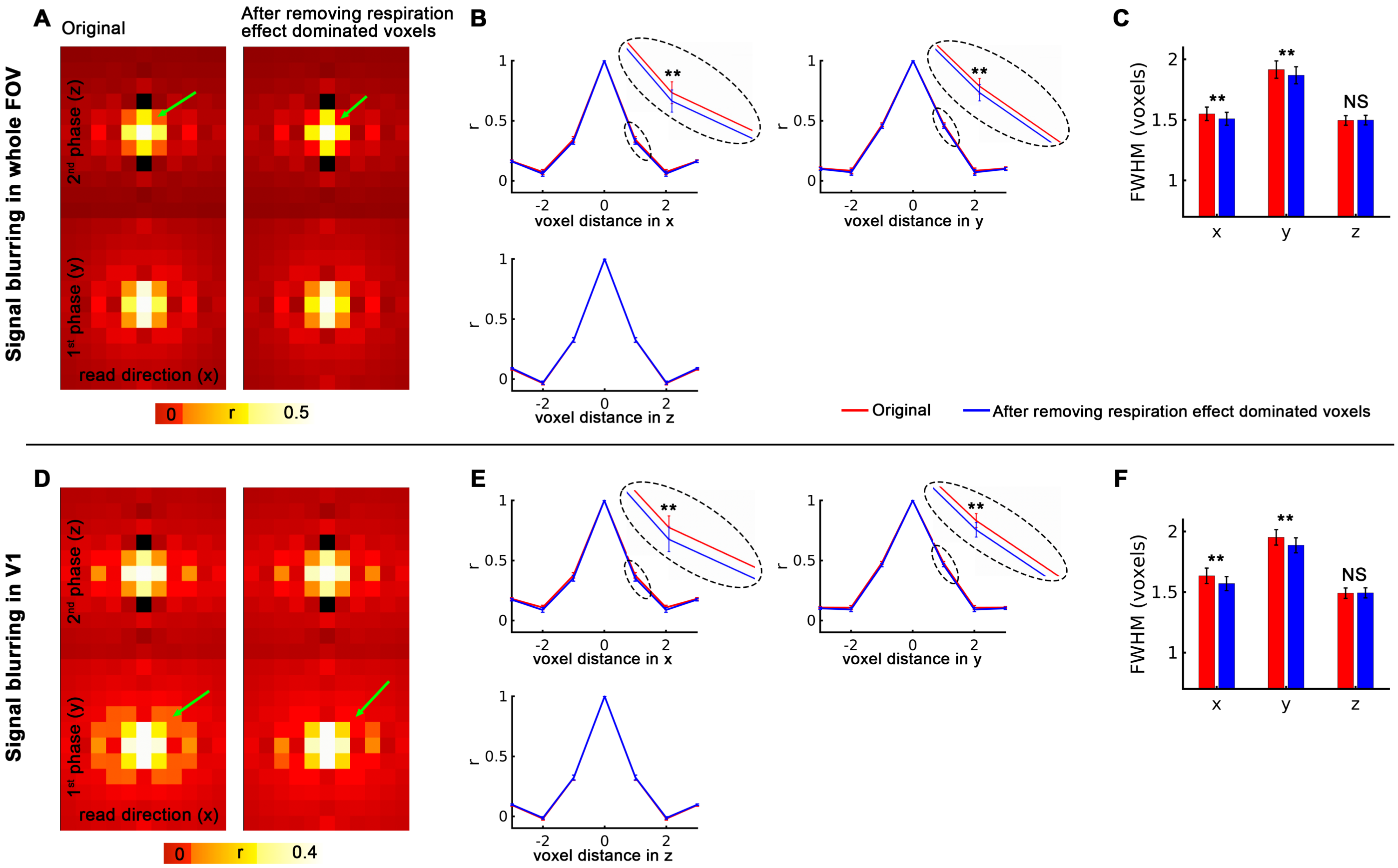

Figure S7. Spatial blurring in VAPER images before and after removing respiration effect dominated voxels. The upper row displays results derived from the brain region of the entire field of view (FOV), while the lower row focuses on the V1 region specifically. **(A) & (D)** depict the blurring kernel, calculated as the mean correlation of each voxel with its neighbors over the whole FOV or V1, respectively, and then averaged across all sessions (N = 9). The center voxel of the kernel has a correlation value of 1, as it is corelated with itself. The correlation values in the blurring kernel after removing respiration effect dominated voxels are lower than that in the original images, indicated by green arrows. **(B) & (E)** display profiles extracted from the signal blurring kernel of VAPER images along each spatial axis (x, y and z) across the center. The correlations with neighboring voxels in the readout and 1st phase encoding direction (highlighted in the dashed circles) are significantly lower after removing respiration effect dominated voxels (paired t-test, ** indicates $p < 0.01$), suggesting less blurring. **(C) & (F)** present bar plots of the full width at half maximum (FWHM) of the blurring kernel in x, y and z directions. Following the exclusion of the voxels most sensitive to respiration effects, the FWHM of the blurring kernel is significantly reduced in both x and y directions (paired t-test, ** indicates $p < 0.01$, NS indicates non-significant). Error bars represent ± SEM across sessions (N = 9).

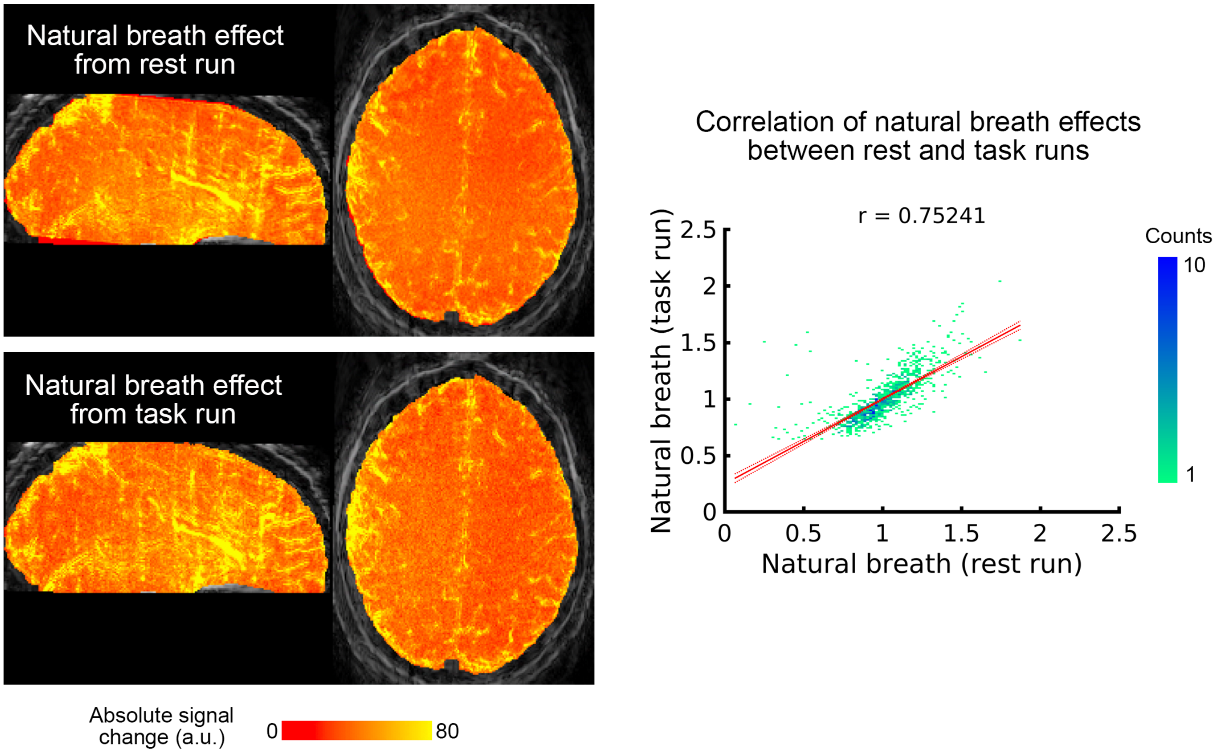

Figure S8. Brain maps of VAPER signal changes induced by natural respiratory variation during resting state and task runs. The right panel displays the node-wise correlation of these natural breath effects on fMRI between rest and task conditions.

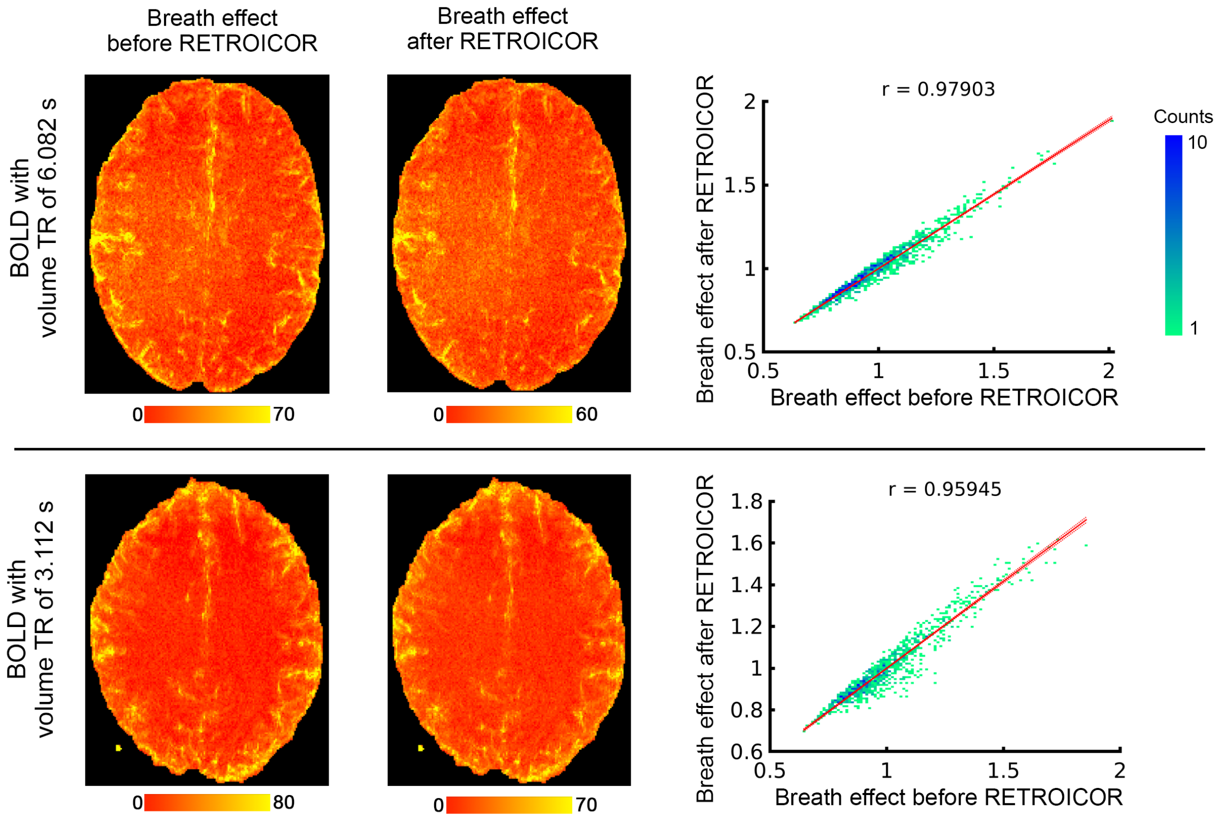

Figure S9. Brain maps of BOLD signal changes induced by natural respiration variation before and after RETROICOR. The upper row displays results from the run with a volume TR of 6.082 s, while the lower row presents results from the run with a volume TR of 3.112 s. The left two columns show the BOLD fMRI response to natural respiratory variations before and after RETROICOR, respectively. The right column shows the node-wise correlation between the respiration effect maps before and after RETROICOR. The high degree of similarity suggests RETROICOR has a minimal effect on altering the detected respiration effects on fMRI, which could be partially attributed to the challenges associated with linear modelling of physiological signals during 3D acquisitions.

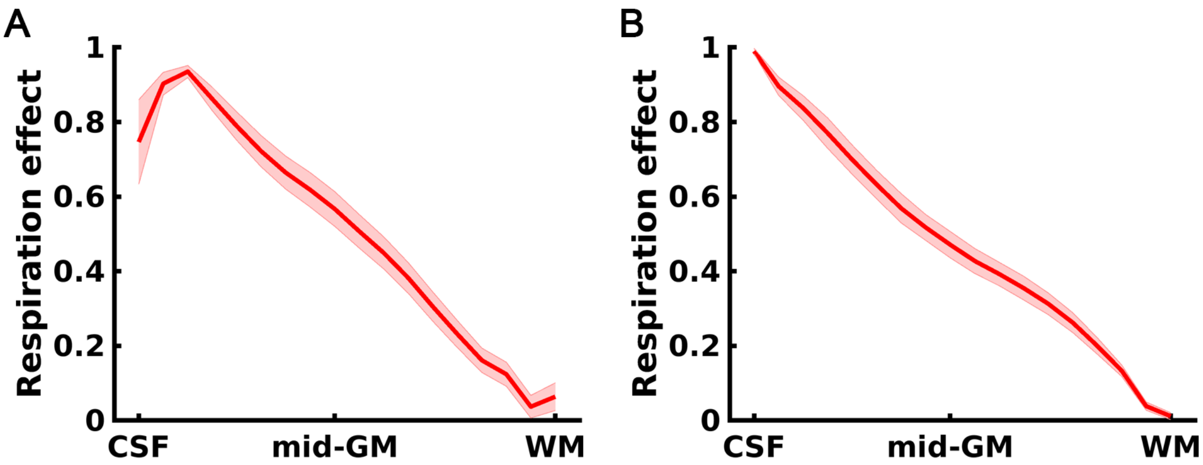

Figure S10. Group-mean laminar profiles of respiration effect on VAPER signal. **(A)** and **(B)** display laminar profiles of respiration effect averaged across the whole cortical areas and V1 area, respectively. On the x-axis, "CSF" indicates the CSF-GM boundary (or cortical surface), "mid-GM" represents the middle cortical depth within GM, and WM denotes the boundary between GM and WM. The respiration effect on the Y-axis is normalized to the 0-1 range before group averaging, for highlighting the profile shape rather than absolute values. Error bars represent ± SEM across individuals. Laminar profile of BOLD respiration effect is very similar to the VAPER profile shown here. It is known that the middle cortical layer has the highest microvascular density in primary sensory and visual cortex (Schmid et al., 2017), which is distinct to the laminar distribution of respiration effects.
